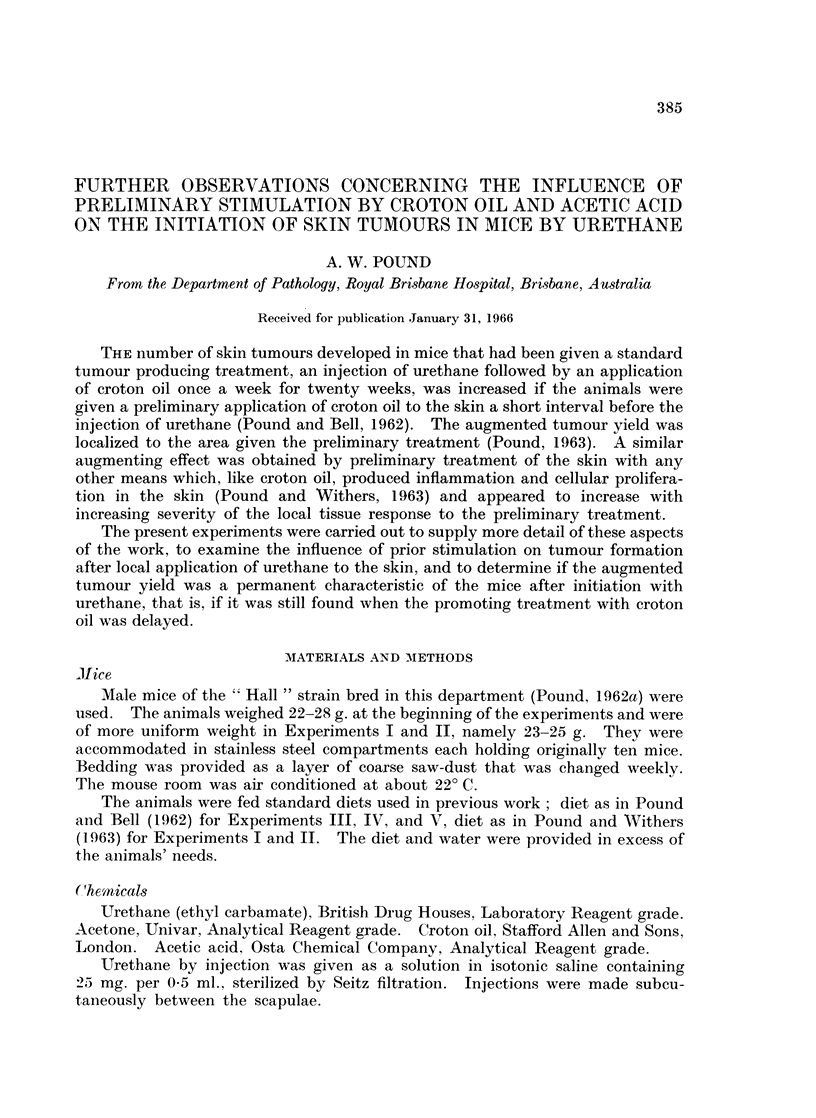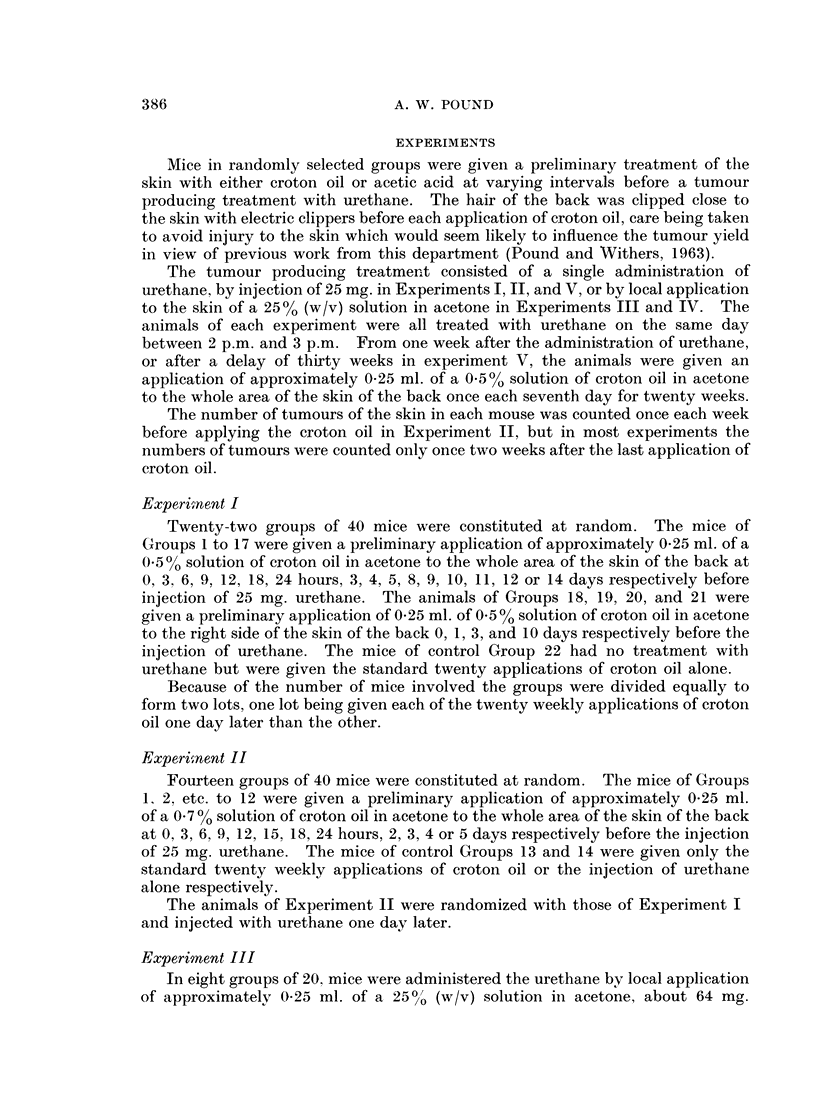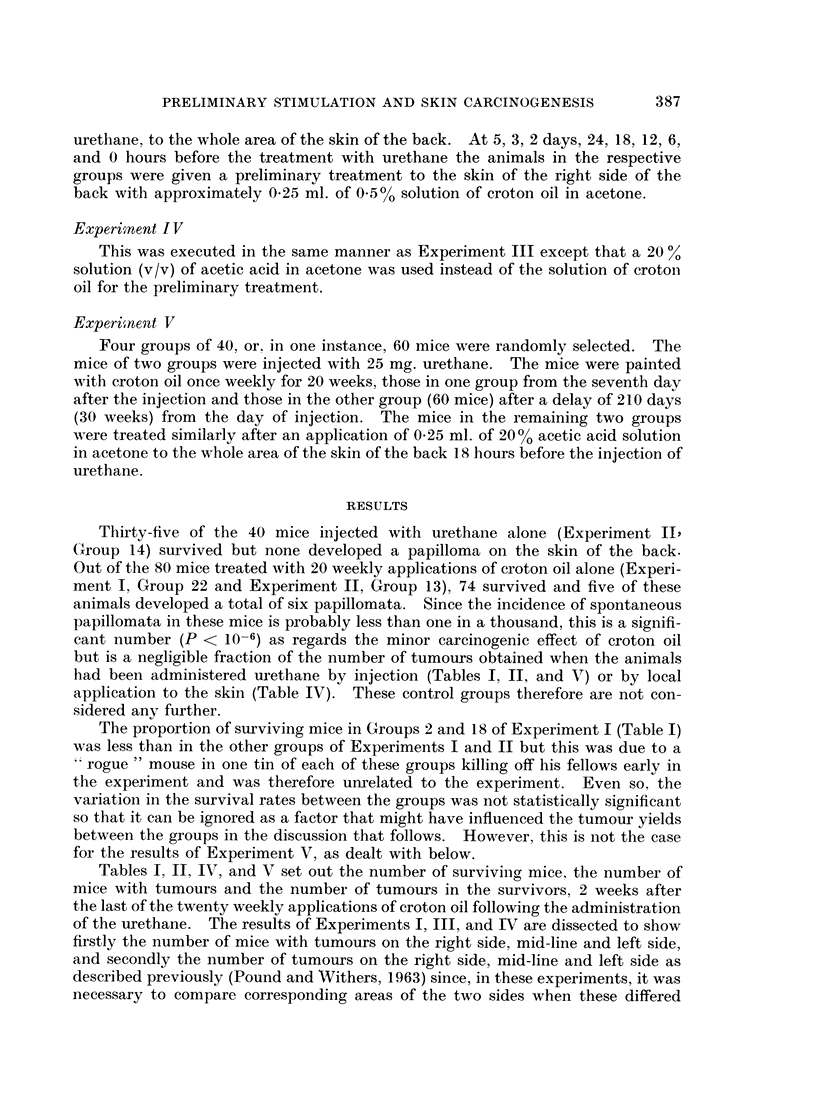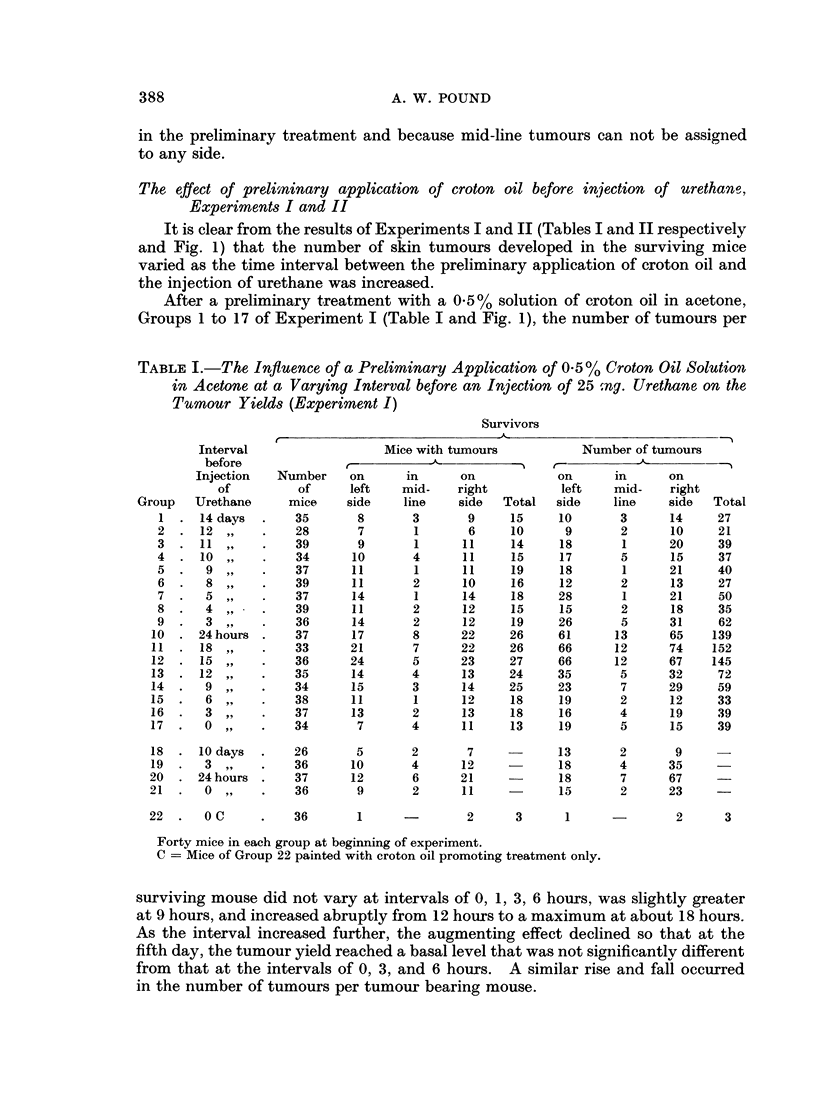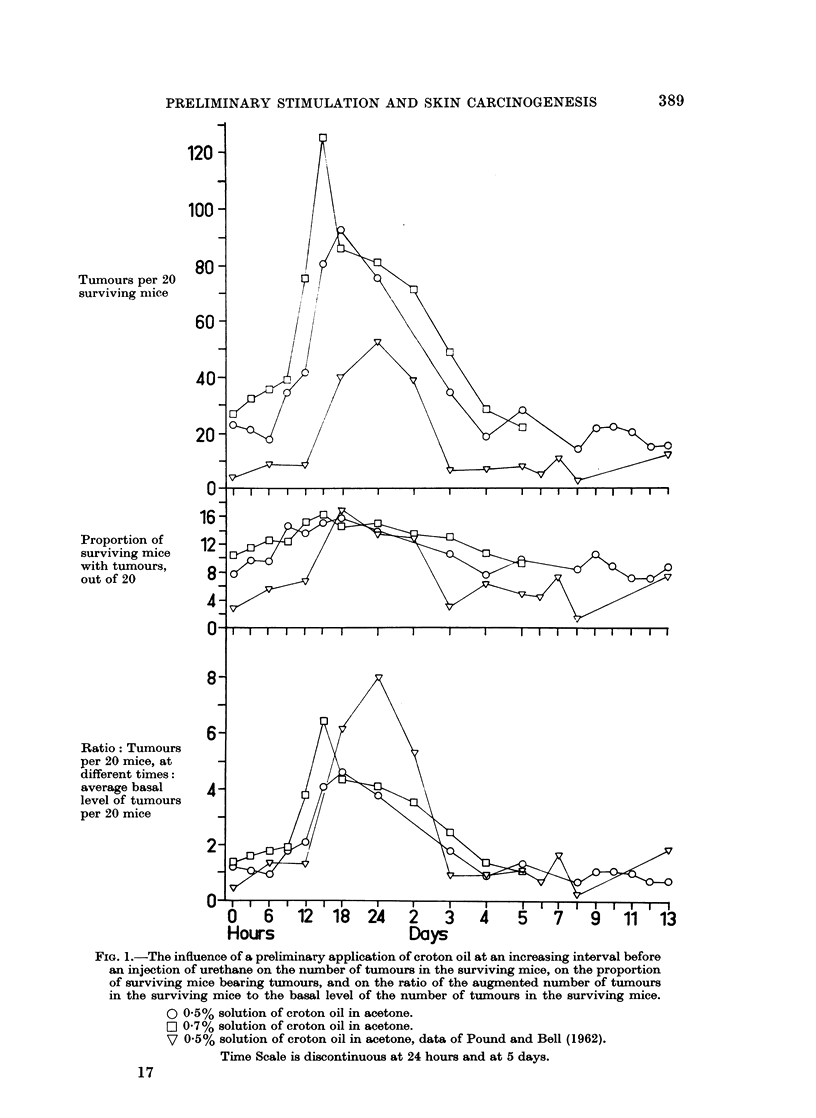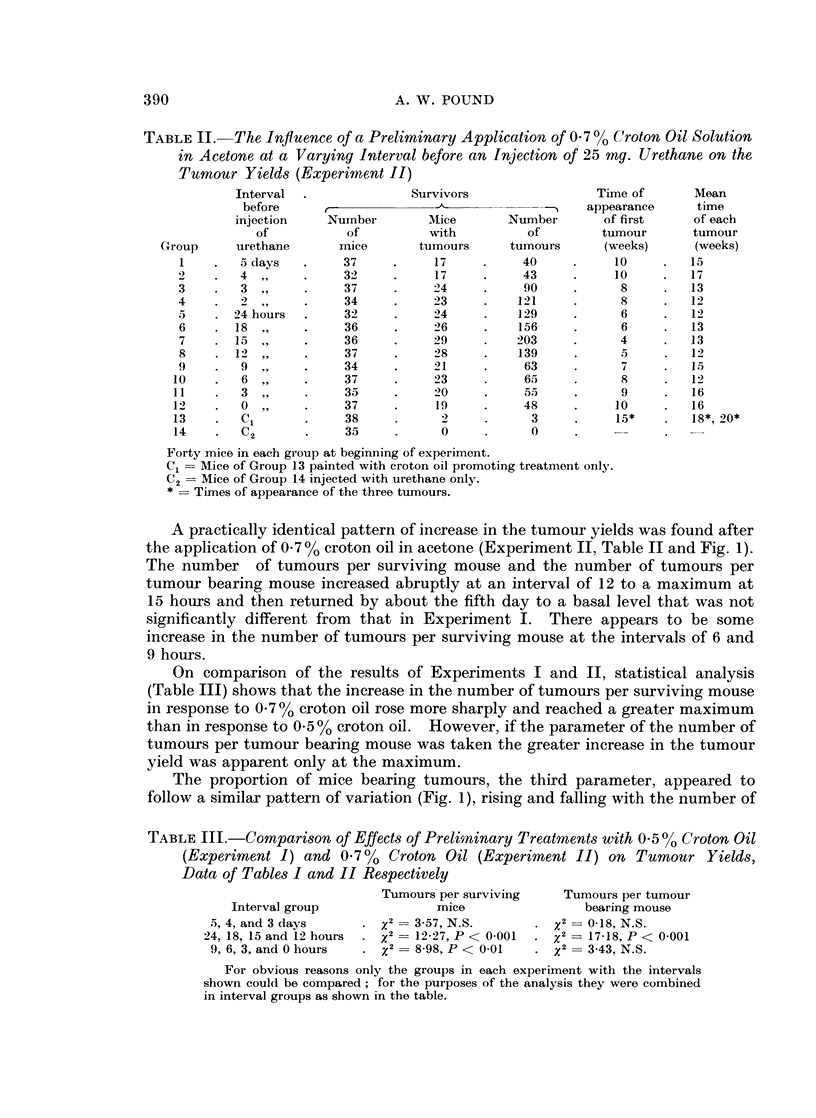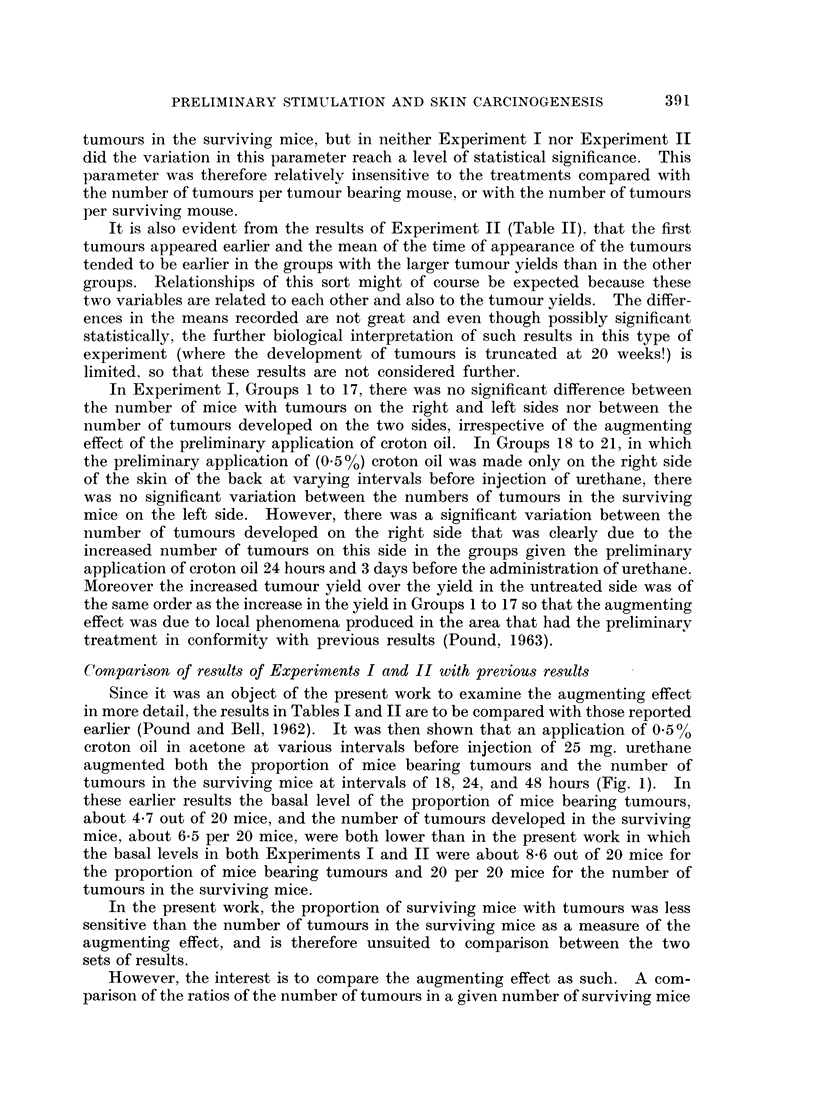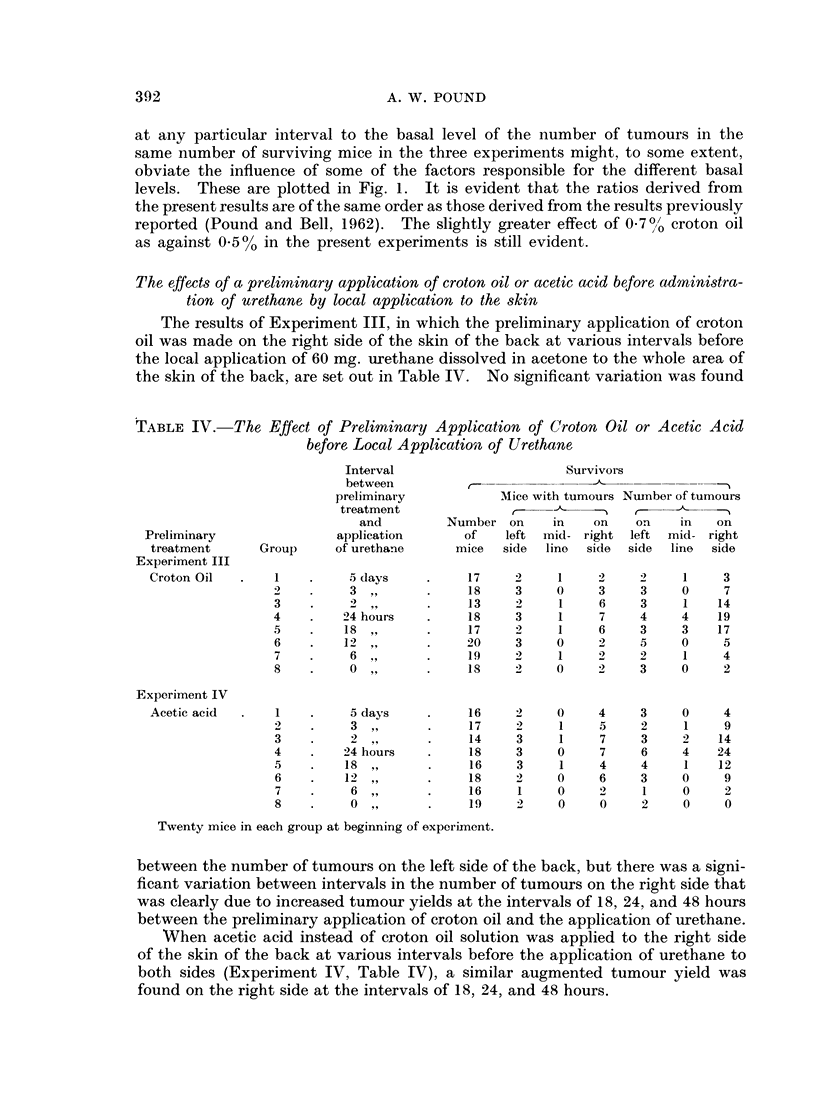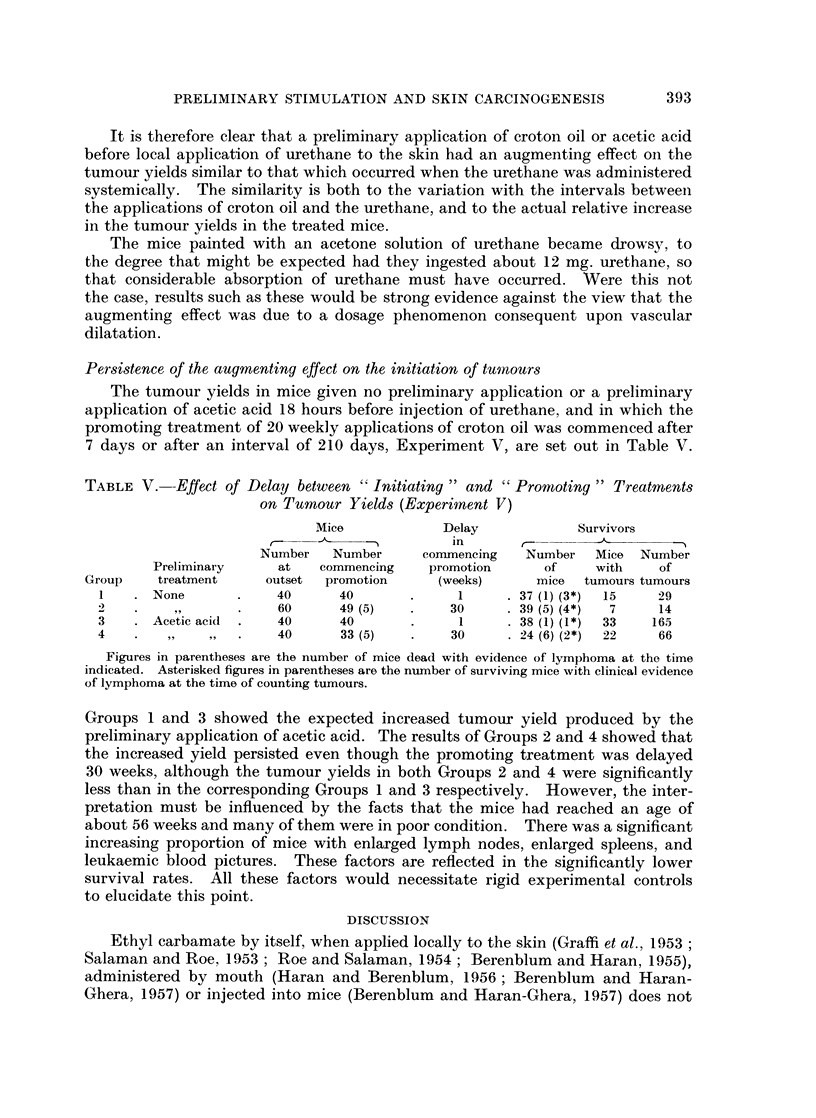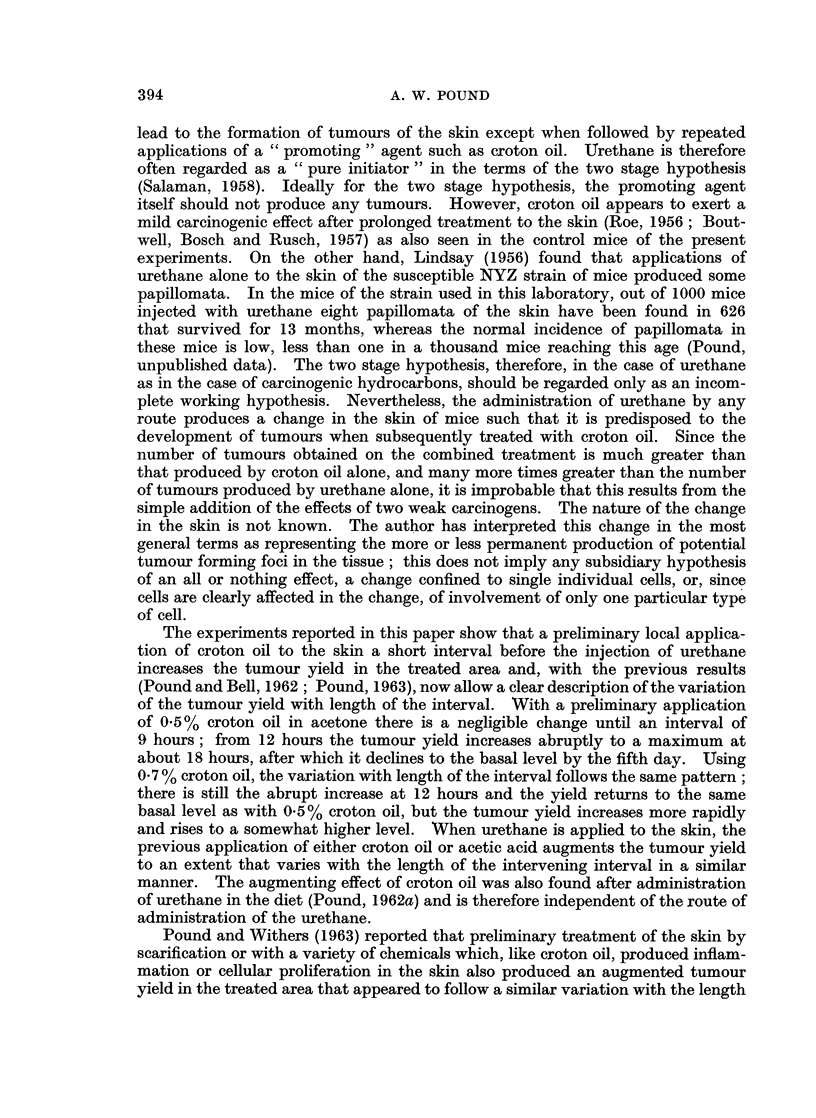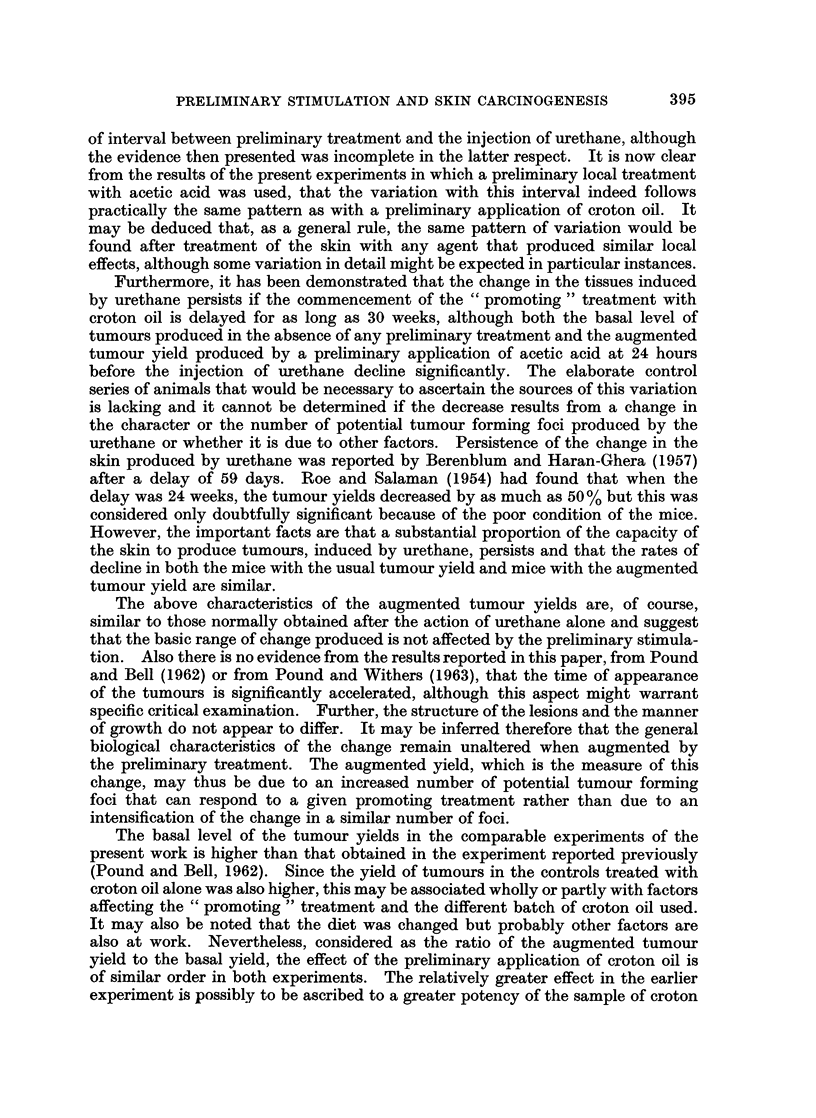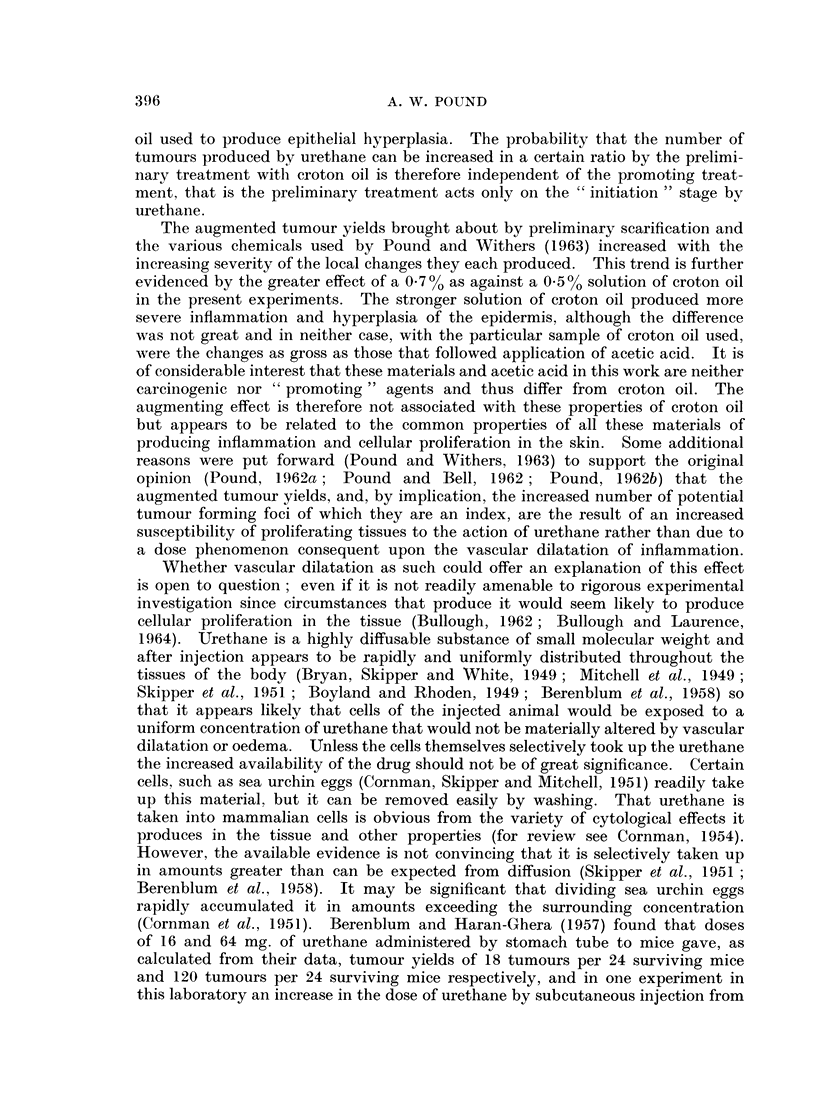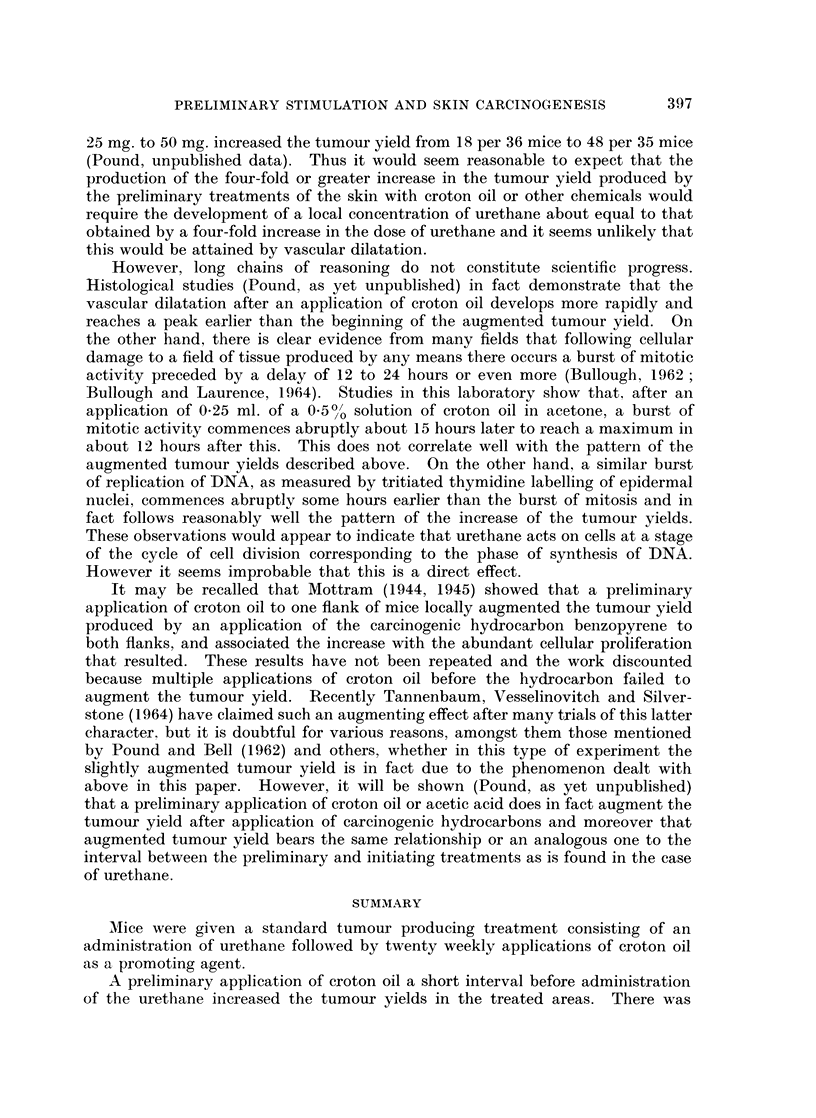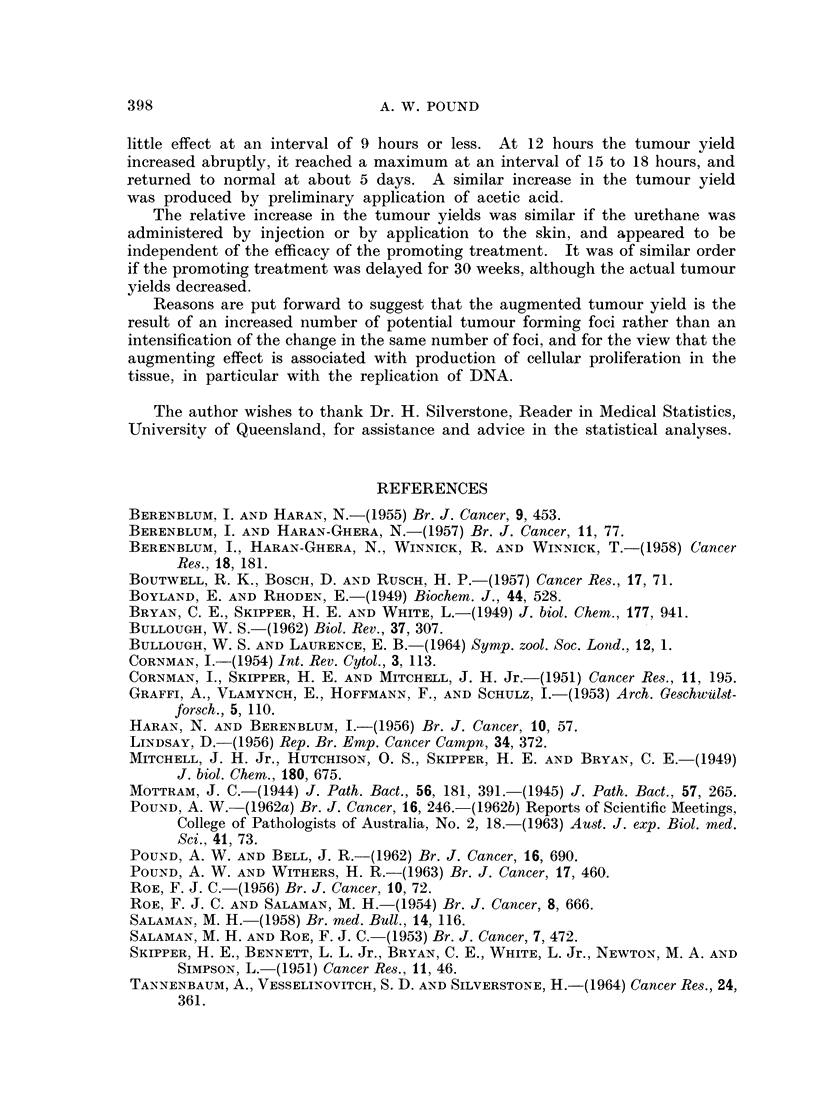# Further observations concerning the influence of preliminary stimulation by croton oil and acetic acid on the initiation of skin tumours in mice by urethane.

**DOI:** 10.1038/bjc.1966.46

**Published:** 1966-06

**Authors:** A. W. Pound


### FURTHER OBSERVATIONS CONCERNING THE INFLUENCE OF PRELIMINARY STIMULATION BY CROTON OIL AND ACETIC ACID ON THE INITIATION OF SKIN TUMOURS IN MICE BY URETHANE

A. W. POUND

*From the Department of Pathology, Royal Brisbane Hospital, Brisbane, Australia*

Received for publication January 31, 1966

THE number of skin tumours developed in mice that had been given a standard tumour producing treatment, an injection of urethane followed by an application of croton oil once a week for twenty weeks, was increased if the animals were given a preliminary application of croton oil to the skin a short interval before the injection of urethane (Pound and Bell, 1962). The augmented tumour yield was localized to the area given the preliminary treatment (Pound, 1963). A similar augmenting effect was obtained by preliminary treatment of the skin with any other means which, like croton oil, produced inflammation and cellular proliferation in the skin (Pound and Withers, 1963) and appeared to increase with increasing severity of the local tissue response to the preliminary treatment.

The present experiments were carried out to supply more detail of these aspects of the work, to examine the influence of prior stimulation on tumour formation after local application of urethane to the skin, and to determine if the augmented tumour yield was a permanent characteristic of the mice after initiation with urethane, that is, if it was still found when the promoting treatment with croton oil was delayed.

### MATERIALS AND METHODS

### *Mice*

Male mice of the " Hall " strain bred in this department (Pound, 1962*a*) were used. The animals weighed 22–28 g. at the beginning of the experiments and were of more uniform weight in Experiments I and II, namely 23–25 g. They were accommodated in stainless steel compartments each holding originally ten mice. Bedding was provided as a layer of coarse saw-dust that was changed weekly. The mouse room was air conditioned at about 22° C.

The animals were fed standard diets used in previous work ; diet as in Pound and Bell (1962) for Experiments III, IV, and V, diet as in Pound and Withers (1963) for Experiments I and II. The diet and water were provided in excess of the animals' needs.

### *Chemicals*

Urethane (ethyl carbamate), British Drug Houses, Laboratory Reagent grade. Acetone, Univar, Analytical Reagent grade. Croton oil, Stafford Allen and Sons, London. Acetic acid, Osta Chemical Company, Analytical Reagent grade.

Urethane by injection was given as a solution in isotonic saline containing 25 mg. per 0·5 ml., sterilized by Seitz filtration. Injections were made subcutaneously between the scapulae.

### EXPERIMENTS

Mice in randomly selected groups were given a preliminary treatment of the skin with either croton oil or acetic acid at varying intervals before a tumour producing treatment with urethane. The hair of the back was clipped close to the skin with electric clippers before each application of croton oil, care being taken to avoid injury to the skin which would seem likely to influence the tumour yield in view of previous work from this department (Pound and Withers, 1963).

The tumour producing treatment consisted of a single administration of urethane, by injection of 25 mg. in Experiments I, II, and V, or by local application to the skin of a 25% (w/v) solution in acetone in Experiments III and IV. The animals of each experiment were all treated with urethane on the same day between 2 p.m. and 3 p.m. From one week after the administration of urethane, or after a delay of thirty weeks in experiment V, the animals were given an application of approximately 0·25 ml. of a 0·5% solution of croton oil in acetone to the whole area of the skin of the back once each seventh day for twenty weeks.

The number of tumours of the skin in each mouse was counted once each week before applying the croton oil in Experiment II, but in most experiments the numbers of tumours were counted only once two weeks after the last application of croton oil.

### *Experiment I*

Twenty-two groups of 40 mice were constituted at random. The mice of Groups 1 to 17 were given a preliminary application of approximately 0·25 ml. of a 0·5% solution of croton oil in acetone to the whole area of the skin of the back at 0, 3, 6, 9, 12, 18, 24 hours, 3, 4, 5, 8, 9, 10, 11, 12 or 14 days respectively before injection of 25 mg. urethane. The animals of Groups 18, 19, 20, and 21 were given a preliminary application of 0·25 ml. of 0·5% solution of croton oil in acetone to the right side of the skin of the back 0, 1, 3, and 10 days respectively before the injection of urethane. The mice of control Group 22 had no treatment with urethane but were given the standard twenty applications of croton oil alone.

Because of the number of mice involved the groups were divided equally to form two lots, one lot being given each of the twenty weekly applications of croton oil one day later than the other.

### *Experiment II*

Fourteen groups of 40 mice were constituted at random. The mice of Groups 1, 2, etc. to 12 were given a preliminary application of approximately 0·25 ml. of a 0·7% solution of croton oil in acetone to the whole area of the skin of the back at 0, 3, 6, 9, 12, 15, 18, 24 hours, 2, 3, 4 or 5 days respectively before the injection of 25 mg. urethane. The mice of control Groups 13 and 14 were given only the standard twenty weekly applications of croton oil or the injection of urethane alone respectively.

The animals of Experiment II were randomized with those of Experiment I and injected with urethane one day later.

### *Experiment III*

In eight groups of 20, mice were administered the urethane by local application of approximately 0·25 ml. of a 25% (w/v) solution in acetone, about 64 mg.

urethane, to the whole area of the skin of the back. At 5, 3, 2 days, 24, 18, 12, 6, and 0 hours before the treatment with urethane the animals in the respective groups were given a preliminary treatment to the skin of the right side of the back with approximately 0·25 ml. of 0·5% solution of croton oil in acetone.

*Experiment IV*

This was executed in the same manner as Experiment III except that a 20% solution (v/v) of acetic acid in acetone was used instead of the solution of croton oil for the preliminary treatment.

*Experiment V*

Four groups of 40, or, in one instance, 60 mice were randomly selected. The mice of two groups were injected with 25 mg. urethane. The mice were painted with croton oil once weekly for 20 weeks, those in one group from the seventh day after the injection and those in the other group (60 mice) after a delay of 210 days (30 weeks) from the day of injection. The mice in the remaining two groups were treated similarly after an application of 0·25 ml. of 20% acetic acid solution in acetone to the whole area of the skin of the back 18 hours before the injection of urethane.

### RESULTS

Thirty-five of the 40 mice injected with urethane alone (Experiment II, Group 14) survived but none developed a papilloma on the skin of the back. Out of the 80 mice treated with 20 weekly applications of croton oil alone (Experiment I, Group 22 and Experiment II, Group 13), 74 survived and five of these animals developed a total of six papillomata. Since the incidence of spontaneous papillomata in these mice is probably less than one in a thousand, this is a significant number ($P < 10^{-6}$) as regards the minor carcinogenic effect of croton oil but is a negligible fraction of the number of tumours obtained when the animals had been administered urethane by injection (Tables I, II, and V) or by local application to the skin (Table IV). These control groups therefore are not considered any further.

The proportion of surviving mice in Groups 2 and 18 of Experiment I (Table I) was less than in the other groups of Experiments I and II but this was due to a " rogue " mouse in one tin of each of these groups killing off his fellows early in the experiment and was therefore unrelated to the experiment. Even so, the variation in the survival rates between the groups was not statistically significant so that it can be ignored as a factor that might have influenced the tumour yields between the groups in the discussion that follows. However, this is not the case for the results of Experiment V, as dealt with below.

Tables I, II, IV, and V set out the number of surviving mice, the number of mice with tumours and the number of tumours in the survivors, 2 weeks after the last of the twenty weekly applications of croton oil following the administration of the urethane. The results of Experiments I, III, and IV are dissected to show firstly the number of mice with tumours on the right side, mid-line and left side, and secondly the number of tumours on the right side, mid-line and left side as described previously (Pound and Withers, 1963) since, in these experiments, it was necessary to compare corresponding areas of the two sides when these differed in the preliminary treatment and because mid-line tumours can not be assigned to any side.

*The effect of preliminary application of croton oil before injection of urethane, Experiments I and II*

It is clear from the results of Experiments I and II (Tables I and II respectively and Fig. 1) that the number of skin tumours developed in the surviving mice varied as the time interval between the preliminary application of croton oil and the injection of urethane was increased.

After a preliminary treatment with a 0·5% solution of croton oil in acetone, Groups 1 to 17 of Experiment I (Table I and Fig. 1), the number of tumours per

TABLE I.—*The Influence of a Preliminary Application of* 0·5% *Croton Oil Solution in Acetone at a Varying Interval before an Injection of* 25 *mg. Urethane on the Tumour Yields* (*Experiment I*)

| | | Survivors | | | | | | | | |
|---|---|---|---|---|---|---|---|---|---|---|
| | | | Mice with tumours | | | | Number of tumours | | | |
| Group | Interval before Injection of Urethane | Number of mice | on left side | in mid-line | on right side | Total | on left side | in mid-line | on right side | Total |
| 1 | 14 days | 35 | 8 | 3 | 9 | 15 | 10 | 3 | 14 | 27 |
| 2 | 12 ,, | 28 | 7 | 1 | 6 | 10 | 9 | 2 | 10 | 21 |
| 3 | 11 ,, | 39 | 9 | 1 | 11 | 14 | 18 | 1 | 20 | 39 |
| 4 | 10 ,, | 34 | 10 | 4 | 11 | 15 | 17 | 5 | 15 | 37 |
| 5 | 9 ,, | 37 | 11 | 1 | 11 | 19 | 18 | 1 | 21 | 40 |
| 6 | 8 ,, | 39 | 11 | 2 | 10 | 16 | 12 | 2 | 13 | 27 |
| 7 | 5 ,, | 37 | 14 | 1 | 14 | 18 | 28 | 1 | 21 | 50 |
| 8 | 4 ,, | 39 | 11 | 2 | 12 | 15 | 15 | 2 | 18 | 35 |
| 9 | 3 ,, | 36 | 14 | 2 | 12 | 19 | 26 | 5 | 31 | 62 |
| 10 | 24 hours | 37 | 17 | 8 | 22 | 26 | 61 | 13 | 65 | 139 |
| 11 | 18 ,, | 33 | 21 | 7 | 22 | 26 | 66 | 12 | 74 | 152 |
| 12 | 15 ,, | 36 | 24 | 5 | 23 | 27 | 66 | 12 | 67 | 145 |
| 13 | 12 ,, | 35 | 14 | 4 | 13 | 24 | 35 | 5 | 32 | 72 |
| 14 | 9 ,, | 34 | 15 | 3 | 14 | 25 | 23 | 7 | 29 | 59 |
| 15 | 6 ,, | 38 | 11 | 1 | 12 | 18 | 19 | 2 | 12 | 33 |
| 16 | 3 ,, | 37 | 13 | 2 | 13 | 18 | 16 | 4 | 19 | 39 |
| 17 | 0 ,, | 34 | 7 | 4 | 11 | 13 | 19 | 5 | 15 | 39 |
| 18 | 10 days | 26 | 5 | 2 | 7 | — | 13 | 2 | 9 | — |
| 19 | 3 ,, | 36 | 10 | 4 | 12 | — | 18 | 4 | 35 | — |
| 20 | 24 hours | 37 | 12 | 6 | 21 | — | 18 | 7 | 67 | — |
| 21 | 0 ,, | 36 | 9 | 2 | 11 | — | 15 | 2 | 23 | — |
| 22 | 0 C | 36 | 1 | — | 2 | 3 | 1 | — | 2 | 3 |

Forty mice in each group at beginning of experiment.
C = Mice of Group 22 painted with croton oil promoting treatment only.

surviving mouse did not vary at intervals of 0, 1, 3, 6 hours, was slightly greater at 9 hours, and increased abruptly from 12 hours to a maximum at about 18 hours. As the interval increased further, the augmenting effect declined so that at the fifth day, the tumour yield reached a basal level that was not significantly different from that at the intervals of 0, 3, and 6 hours. A similar rise and fall occurred in the number of tumours per tumour bearing mouse.

FIG. 1.—The influence of a preliminary application of croton oil at an increasing interval before an injection of urethane on the number of tumours in the surviving mice, on the proportion of surviving mice bearing tumours, and on the ratio of the augmented number of tumours in the surviving mice to the basal level of the number of tumours in the surviving mice.

○ 0·5% solution of croton oil in acetone.
□ 0·7% solution of croton oil in acetone.
▽ 0·5% solution of croton oil in acetone, data of Pound and Bell (1962).

Time Scale is discontinuous at 24 hours and at 5 days.

TABLE II.—*The Influence of a Preliminary Application of* 0·7 % *Croton Oil Solution in Acetone at a Varying Interval before an Injection of* 25 *mg. Urethane on the Tumour Yields* (*Experiment II*)

| Group | Interval before injection of urethane | Survivors: Number of mice | Survivors: Mice with tumours | Survivors: Number of tumours | Time of appearance of first tumour (weeks) | Mean time of each tumour (weeks) |
|---|---|---|---|---|---|---|
| 1 | 5 days | 37 | 17 | 40 | 10 | 15 |
| 2 | 4 ,, | 32 | 17 | 43 | 10 | 17 |
| 3 | 3 ,, | 37 | 24 | 90 | 8 | 13 |
| 4 | 2 ,, | 34 | 23 | 121 | 8 | 12 |
| 5 | 24 hours | 32 | 24 | 129 | 6 | 12 |
| 6 | 18 ,, | 36 | 26 | 156 | 6 | 13 |
| 7 | 15 ,, | 36 | 29 | 203 | 4 | 13 |
| 8 | 12 ,, | 37 | 28 | 139 | 5 | 12 |
| 9 | 9 ,, | 34 | 21 | 63 | 7 | 15 |
| 10 | 6 ,, | 37 | 23 | 65 | 8 | 12 |
| 11 | 3 ,, | 35 | 20 | 55 | 9 | 16 |
| 12 | 0 ,, | 37 | 19 | 48 | 10 | 16 |
| 13 | $C_1$ | 38 | 2 | 3 | 15* | 18*, 20* |
| 14 | $C_2$ | 35 | 0 | 0 | — | — |

Forty mice in each group at beginning of experiment.
$C_1$ = Mice of Group 13 painted with croton oil promoting treatment only.
$C_2$ = Mice of Group 14 injected with urethane only.
* = Times of appearance of the three tumours.

A practically identical pattern of increase in the tumour yields was found after the application of 0·7 % croton oil in acetone (Experiment II, Table II and Fig. 1). The number of tumours per surviving mouse and the number of tumours per tumour bearing mouse increased abruptly at an interval of 12 to a maximum at 15 hours and then returned by about the fifth day to a basal level that was not significantly different from that in Experiment I. There appears to be some increase in the number of tumours per surviving mouse at the intervals of 6 and 9 hours.

On comparison of the results of Experiments I and II, statistical analysis (Table III) shows that the increase in the number of tumours per surviving mouse in response to 0·7 % croton oil rose more sharply and reached a greater maximum than in response to 0·5 % croton oil. However, if the parameter of the number of tumours per tumour bearing mouse was taken the greater increase in the tumour yield was apparent only at the maximum.

The proportion of mice bearing tumours, the third parameter, appeared to follow a similar pattern of variation (Fig. 1), rising and falling with the number of

TABLE III.—*Comparison of Effects of Preliminary Treatments with* 0·5 % *Croton Oil* (*Experiment I*) *and* 0·7 % *Croton Oil* (*Experiment II*) *on Tumour Yields, Data of Tables I and II Respectively*

| Interval group | Tumours per surviving mice | Tumours per tumour bearing mouse |
|---|---|---|
| 5, 4, and 3 days | $\chi^2 = 3{\cdot}57$, N.S. | $\chi^2 = 0{\cdot}18$, N.S. |
| 24, 18, 15 and 12 hours | $\chi^2 = 12{\cdot}27$, $P < 0{\cdot}001$ | $\chi^2 = 17{\cdot}18$, $P < 0{\cdot}001$ |
| 9, 6, 3, and 0 hours | $\chi^2 = 8{\cdot}98$, $P < 0{\cdot}01$ | $\chi^2 = 3{\cdot}43$, N.S. |

For obvious reasons only the groups in each experiment with the intervals shown could be compared ; for the purposes of the analysis they were combined in interval groups as shown in the table.

tumours in the surviving mice, but in neither Experiment I nor Experiment II did the variation in this parameter reach a level of statistical significance. This parameter was therefore relatively insensitive to the treatments compared with the number of tumours per tumour bearing mouse, or with the number of tumours per surviving mouse.

It is also evident from the results of Experiment II (Table II), that the first tumours appeared earlier and the mean of the time of appearance of the tumours tended to be earlier in the groups with the larger tumour yields than in the other groups. Relationships of this sort might of course be expected because these two variables are related to each other and also to the tumour yields. The differences in the means recorded are not great and even though possibly significant statistically, the further biological interpretation of such results in this type of experiment (where the development of tumours is truncated at 20 weeks!) is limited, so that these results are not considered further.

In Experiment I, Groups 1 to 17, there was no significant difference between the number of mice with tumours on the right and left sides nor between the number of tumours developed on the two sides, irrespective of the augmenting effect of the preliminary application of croton oil. In Groups 18 to 21, in which the preliminary application of (0·5%) croton oil was made only on the right side of the skin of the back at varying intervals before injection of urethane, there was no significant variation between the numbers of tumours in the surviving mice on the left side. However, there was a significant variation between the number of tumours developed on the right side that was clearly due to the increased number of tumours on this side in the groups given the preliminary application of croton oil 24 hours and 3 days before the administration of urethane. Moreover the increased tumour yield over the yield in the untreated side was of the same order as the increase in the yield in Groups 1 to 17 so that the augmenting effect was due to local phenomena produced in the area that had the preliminary treatment in conformity with previous results (Pound, 1963).

*Comparison of results of Experiments I and II with previous results*

Since it was an object of the present work to examine the augmenting effect in more detail, the results in Tables I and II are to be compared with those reported earlier (Pound and Bell, 1962). It was then shown that an application of 0·5% croton oil in acetone at various intervals before injection of 25 mg. urethane augmented both the proportion of mice bearing tumours and the number of tumours in the surviving mice at intervals of 18, 24, and 48 hours (Fig. 1). In these earlier results the basal level of the proportion of mice bearing tumours, about 4·7 out of 20 mice, and the number of tumours developed in the surviving mice, about 6·5 per 20 mice, were both lower than in the present work in which the basal levels in both Experiments I and II were about 8·6 out of 20 mice for the proportion of mice bearing tumours and 20 per 20 mice for the number of tumours in the surviving mice.

In the present work, the proportion of surviving mice with tumours was less sensitive than the number of tumours in the surviving mice as a measure of the augmenting effect, and is therefore unsuited to comparison between the two sets of results.

However, the interest is to compare the augmenting effect as such. A comparison of the ratios of the number of tumours in a given number of surviving mice at any particular interval to the basal level of the number of tumours in the same number of surviving mice in the three experiments might, to some extent, obviate the influence of some of the factors responsible for the different basal levels. These are plotted in Fig. 1. It is evident that the ratios derived from the present results are of the same order as those derived from the results previously reported (Pound and Bell, 1962). The slightly greater effect of 0·7% croton oil as against 0·5% in the present experiments is still evident.

*The effects of a preliminary application of croton oil or acetic acid before administration of urethane by local application to the skin*

The results of Experiment III, in which the preliminary application of croton oil was made on the right side of the skin of the back at various intervals before the local application of 60 mg. urethane dissolved in acetone to the whole area of the skin of the back, are set out in Table IV. No significant variation was found

TABLE IV.—*The Effect of Preliminary Application of Croton Oil or Acetic Acid before Local Application of Urethane*

| Preliminary treatment | Group | Interval between preliminary treatment and application of urethane | Survivors | | | | | | |
|---|---|---|---|---|---|---|---|---|---|
| | | | | Mice with tumours | | | Number of tumours | | |
| | | | Number of mice | on left side | in mid-line | on right side | on left side | in mid-line | on right side |
| Experiment III | | | | | | | | | |
| Croton Oil | 1 | 5 days | 17 | 2 | 1 | 2 | 2 | 1 | 3 |
| | 2 | 3 ,, | 18 | 3 | 0 | 3 | 3 | 0 | 7 |
| | 3 | 2 ,, | 13 | 2 | 1 | 6 | 3 | 1 | 14 |
| | 4 | 24 hours | 18 | 3 | 1 | 7 | 4 | 4 | 19 |
| | 5 | 18 ,, | 17 | 2 | 1 | 6 | 3 | 3 | 17 |
| | 6 | 12 ,, | 20 | 3 | 0 | 2 | 5 | 0 | 5 |
| | 7 | 6 ,, | 19 | 2 | 1 | 2 | 2 | 1 | 4 |
| | 8 | 0 ,, | 18 | 2 | 0 | 2 | 3 | 0 | 2 |
| Experiment IV | | | | | | | | | |
| Acetic acid | 1 | 5 days | 16 | 2 | 0 | 4 | 3 | 0 | 4 |
| | 2 | 3 ,, | 17 | 2 | 1 | 5 | 2 | 1 | 9 |
| | 3 | 2 ,, | 14 | 3 | 1 | 7 | 3 | 2 | 14 |
| | 4 | 24 hours | 18 | 3 | 0 | 7 | 6 | 4 | 24 |
| | 5 | 18 ,, | 16 | 3 | 1 | 4 | 4 | 1 | 12 |
| | 6 | 12 ,, | 18 | 2 | 0 | 6 | 3 | 0 | 9 |
| | 7 | 6 ,, | 16 | 1 | 0 | 2 | 1 | 0 | 2 |
| | 8 | 0 ,, | 19 | 2 | 0 | 0 | 2 | 0 | 0 |

Twenty mice in each group at beginning of experiment.

between the number of tumours on the left side of the back, but there was a significant variation between intervals in the number of tumours on the right side that was clearly due to increased tumour yields at the intervals of 18, 24, and 48 hours between the preliminary application of croton oil and the application of urethane.

When acetic acid instead of croton oil solution was applied to the right side of the skin of the back at various intervals before the application of urethane to both sides (Experiment IV, Table IV), a similar augmented tumour yield was found on the right side at the intervals of 18, 24, and 48 hours.

It is therefore clear that a preliminary application of croton oil or acetic acid before local application of urethane to the skin had an augmenting effect on the tumour yields similar to that which occurred when the urethane was administered systemically. The similarity is both to the variation with the intervals between the applications of croton oil and the urethane, and to the actual relative increase in the tumour yields in the treated mice.

The mice painted with an acetone solution of urethane became drowsy, to the degree that might be expected had they ingested about 12 mg. urethane, so that considerable absorption of urethane must have occurred. Were this not the case, results such as these would be strong evidence against the view that the augmenting effect was due to a dosage phenomenon consequent upon vascular dilatation.

*Persistence of the augmenting effect on the initiation of tumours*

The tumour yields in mice given no preliminary application or a preliminary application of acetic acid 18 hours before injection of urethane, and in which the promoting treatment of 20 weekly applications of croton oil was commenced after 7 days or after an interval of 210 days, Experiment V, are set out in Table V.

TABLE V.—*Effect of Delay between "Initiating" and "Promoting" Treatments on Tumour Yields (Experiment V)*

| | | Mice | | Delay in | Survivors | | |
|---|---|---|---|---|---|---|---|
| Group | Preliminary treatment | Number at outset | Number commencing promotion | commencing promotion (weeks) | Number of mice | Mice with tumours | Number of tumours |
| 1 | None | 40 | 40 | 1 | 37 (1) (3*) | 15 | 29 |
| 2 | ,, | 60 | 49 (5) | 30 | 39 (5) (4*) | 7 | 14 |
| 3 | Acetic acid | 40 | 40 | 1 | 38 (1) (1*) | 33 | 165 |
| 4 | ,, ,, | 40 | 33 (5) | 30 | 24 (6) (2*) | 22 | 66 |

Figures in parentheses are the number of mice dead with evidence of lymphoma at the time indicated. Asterisked figures in parentheses are the number of surviving mice with clinical evidence of lymphoma at the time of counting tumours.

Groups 1 and 3 showed the expected increased tumour yield produced by the preliminary application of acetic acid. The results of Groups 2 and 4 showed that the increased yield persisted even though the promoting treatment was delayed 30 weeks, although the tumour yields in both Groups 2 and 4 were significantly less than in the corresponding Groups 1 and 3 respectively. However, the interpretation must be influenced by the facts that the mice had reached an age of about 56 weeks and many of them were in poor condition. There was a significant increasing proportion of mice with enlarged lymph nodes, enlarged spleens, and leukaemic blood pictures. These factors are reflected in the significantly lower survival rates. All these factors would necessitate rigid experimental controls to elucidate this point.

### DISCUSSION

Ethyl carbamate by itself, when applied locally to the skin (Graffi *et al.*, 1953 ; Salaman and Roe, 1953 ; Roe and Salaman, 1954 ; Berenblum and Haran, 1955), administered by mouth (Haran and Berenblum, 1956 ; Berenblum and Haran-Ghera, 1957) or injected into mice (Berenblum and Haran-Ghera, 1957) does not lead to the formation of tumours of the skin except when followed by repeated applications of a " promoting " agent such as croton oil. Urethane is therefore often regarded as a " pure initiator " in the terms of the two stage hypothesis (Salaman, 1958). Ideally for the two stage hypothesis, the promoting agent itself should not produce any tumours. However, croton oil appears to exert a mild carcinogenic effect after prolonged treatment to the skin (Roe, 1956 ; Boutwell, Bosch and Rusch, 1957) as also seen in the control mice of the present experiments. On the other hand, Lindsay (1956) found that applications of urethane alone to the skin of the susceptible NYZ strain of mice produced some papillomata. In the mice of the strain used in this laboratory, out of 1000 mice injected with urethane eight papillomata of the skin have been found in 626 that survived for 13 months, whereas the normal incidence of papillomata in these mice is low, less than one in a thousand mice reaching this age (Pound, unpublished data). The two stage hypothesis, therefore, in the case of urethane as in the case of carcinogenic hydrocarbons, should be regarded only as an incomplete working hypothesis. Nevertheless, the administration of urethane by any route produces a change in the skin of mice such that it is predisposed to the development of tumours when subsequently treated with croton oil. Since the number of tumours obtained on the combined treatment is much greater than that produced by croton oil alone, and many more times greater than the number of tumours produced by urethane alone, it is improbable that this results from the simple addition of the effects of two weak carcinogens. The nature of the change in the skin is not known. The author has interpreted this change in the most general terms as representing the more or less permanent production of potential tumour forming foci in the tissue ; this does not imply any subsidiary hypothesis of an all or nothing effect, a change confined to single individual cells, or, since cells are clearly affected in the change, of involvement of only one particular type of cell.

The experiments reported in this paper show that a preliminary local application of croton oil to the skin a short interval before the injection of urethane increases the tumour yield in the treated area and, with the previous results (Pound and Bell, 1962 ; Pound, 1963), now allow a clear description of the variation of the tumour yield with length of the interval. With a preliminary application of 0·5% croton oil in acetone there is a negligible change until an interval of 9 hours ; from 12 hours the tumour yield increases abruptly to a maximum at about 18 hours, after which it declines to the basal level by the fifth day. Using 0·7% croton oil, the variation with length of the interval follows the same pattern ; there is still the abrupt increase at 12 hours and the yield returns to the same basal level as with 0·5% croton oil, but the tumour yield increases more rapidly and rises to a somewhat higher level. When urethane is applied to the skin, the previous application of either croton oil or acetic acid augments the tumour yield to an extent that varies with the length of the intervening interval in a similar manner. The augmenting effect of croton oil was also found after administration of urethane in the diet (Pound, 1962*a*) and is therefore independent of the route of administration of the urethane.

Pound and Withers (1963) reported that preliminary treatment of the skin by scarification or with a variety of chemicals which, like croton oil, produced inflammation or cellular proliferation in the skin also produced an augmented tumour yield in the treated area that appeared to follow a similar variation with the length of interval between preliminary treatment and the injection of urethane, although the evidence then presented was incomplete in the latter respect. It is now clear from the results of the present experiments in which a preliminary local treatment with acetic acid was used, that the variation with this interval indeed follows practically the same pattern as with a preliminary application of croton oil. It may be deduced that, as a general rule, the same pattern of variation would be found after treatment of the skin with any agent that produced similar local effects, although some variation in detail might be expected in particular instances.

Furthermore, it has been demonstrated that the change in the tissues induced by urethane persists if the commencement of the " promoting " treatment with croton oil is delayed for as long as 30 weeks, although both the basal level of tumours produced in the absence of any preliminary treatment and the augmented tumour yield produced by a preliminary application of acetic acid at 24 hours before the injection of urethane decline significantly. The elaborate control series of animals that would be necessary to ascertain the sources of this variation is lacking and it cannot be determined if the decrease results from a change in the character or the number of potential tumour forming foci produced by the urethane or whether it is due to other factors. Persistence of the change in the skin produced by urethane was reported by Berenblum and Haran-Ghera (1957) after a delay of 59 days. Roe and Salaman (1954) had found that when the delay was 24 weeks, the tumour yields decreased by as much as 50% but this was considered only doubtfully significant because of the poor condition of the mice. However, the important facts are that a substantial proportion of the capacity of the skin to produce tumours, induced by urethane, persists and that the rates of decline in both the mice with the usual tumour yield and mice with the augmented tumour yield are similar.

The above characteristics of the augmented tumour yields are, of course, similar to those normally obtained after the action of urethane alone and suggest that the basic range of change produced is not affected by the preliminary stimulation. Also there is no evidence from the results reported in this paper, from Pound and Bell (1962) or from Pound and Withers (1963), that the time of appearance of the tumours is significantly accelerated, although this aspect might warrant specific critical examination. Further, the structure of the lesions and the manner of growth do not appear to differ. It may be inferred therefore that the general biological characteristics of the change remain unaltered when augmented by the preliminary treatment. The augmented yield, which is the measure of this change, may thus be due to an increased number of potential tumour forming foci that can respond to a given promoting treatment rather than due to an intensification of the change in a similar number of foci.

The basal level of the tumour yields in the comparable experiments of the present work is higher than that obtained in the experiment reported previously (Pound and Bell, 1962). Since the yield of tumours in the controls treated with croton oil alone was also higher, this may be associated wholly or partly with factors affecting the " promoting " treatment and the different batch of croton oil used. It may also be noted that the diet was changed but probably other factors are also at work. Nevertheless, considered as the ratio of the augmented tumour yield to the basal yield, the effect of the preliminary application of croton oil is of similar order in both experiments. The relatively greater effect in the earlier experiment is possibly to be ascribed to a greater potency of the sample of croton oil used to produce epithelial hyperplasia. The probability that the number of tumours produced by urethane can be increased in a certain ratio by the preliminary treatment with croton oil is therefore independent of the promoting treatment, that is the preliminary treatment acts only on the " initiation " stage by urethane.

The augmented tumour yields brought about by preliminary scarification and the various chemicals used by Pound and Withers (1963) increased with the increasing severity of the local changes they each produced. This trend is further evidenced by the greater effect of a 0·7% as against a 0·5% solution of croton oil in the present experiments. The stronger solution of croton oil produced more severe inflammation and hyperplasia of the epidermis, although the difference was not great and in neither case, with the particular sample of croton oil used, were the changes as gross as those that followed application of acetic acid. It is of considerable interest that these materials and acetic acid in this work are neither carcinogenic nor " promoting " agents and thus differ from croton oil. The augmenting effect is therefore not associated with these properties of croton oil but appears to be related to the common properties of all these materials of producing inflammation and cellular proliferation in the skin. Some additional reasons were put forward (Pound and Withers, 1963) to support the original opinion (Pound, 1962*a* ; Pound and Bell, 1962 ; Pound, 1962*b*) that the augmented tumour yields, and, by implication, the increased number of potential tumour forming foci of which they are an index, are the result of an increased susceptibility of proliferating tissues to the action of urethane rather than due to a dose phenomenon consequent upon the vascular dilatation of inflammation.

Whether vascular dilatation as such could offer an explanation of this effect is open to question ; even if it is not readily amenable to rigorous experimental investigation since circumstances that produce it would seem likely to produce cellular proliferation in the tissue (Bullough, 1962 ; Bullough and Laurence, 1964). Urethane is a highly diffusable substance of small molecular weight and after injection appears to be rapidly and uniformly distributed throughout the tissues of the body (Bryan, Skipper and White, 1949 ; Mitchell *et al.*, 1949 ; Skipper *et al.*, 1951 ; Boyland and Rhoden, 1949 ; Berenblum *et al.*, 1958) so that it appears likely that cells of the injected animal would be exposed to a uniform concentration of urethane that would not be materially altered by vascular dilatation or oedema. Unless the cells themselves selectively took up the urethane the increased availability of the drug should not be of great significance. Certain cells, such as sea urchin eggs (Cornman, Skipper and Mitchell, 1951) readily take up this material, but it can be removed easily by washing. That urethane is taken into mammalian cells is obvious from the variety of cytological effects it produces in the tissue and other properties (for review see Cornman, 1954). However, the available evidence is not convincing that it is selectively taken up in amounts greater than can be expected from diffusion (Skipper *et al.*, 1951 ; Berenblum *et al.*, 1958). It may be significant that dividing sea urchin eggs rapidly accumulated it in amounts exceeding the surrounding concentration (Cornman *et al.*, 1951). Berenblum and Haran-Ghera (1957) found that doses of 16 and 64 mg. of urethane administered by stomach tube to mice gave, as calculated from their data, tumour yields of 18 tumours per 24 surviving mice and 120 tumours per 24 surviving mice respectively, and in one experiment in this laboratory an increase in the dose of urethane by subcutaneous injection from

25 mg. to 50 mg. increased the tumour yield from 18 per 36 mice to 48 per 35 mice (Pound, unpublished data). Thus it would seem reasonable to expect that the production of the four-fold or greater increase in the tumour yield produced by the preliminary treatments of the skin with croton oil or other chemicals would require the development of a local concentration of urethane about equal to that obtained by a four-fold increase in the dose of urethane and it seems unlikely that this would be attained by vascular dilatation.

However, long chains of reasoning do not constitute scientific progress. Histological studies (Pound, as yet unpublished) in fact demonstrate that the vascular dilatation after an application of croton oil develops more rapidly and reaches a peak earlier than the beginning of the augmented tumour yield. On the other hand, there is clear evidence from many fields that following cellular damage to a field of tissue produced by any means there occurs a burst of mitotic activity preceded by a delay of 12 to 24 hours or even more (Bullough, 1962 ; Bullough and Laurence, 1964). Studies in this laboratory show that, after an application of 0·25 ml. of a 0·5% solution of croton oil in acetone, a burst of mitotic activity commences abruptly about 15 hours later to reach a maximum in about 12 hours after this. This does not correlate well with the pattern of the augmented tumour yields described above. On the other hand, a similar burst of replication of DNA, as measured by tritiated thymidine labelling of epidermal nuclei, commences abruptly some hours earlier than the burst of mitosis and in fact follows reasonably well the pattern of the increase of the tumour yields. These observations would appear to indicate that urethane acts on cells at a stage of the cycle of cell division corresponding to the phase of synthesis of DNA. However it seems improbable that this is a direct effect.

It may be recalled that Mottram (1944, 1945) showed that a preliminary application of croton oil to one flank of mice locally augmented the tumour yield produced by an application of the carcinogenic hydrocarbon benzopyrene to both flanks, and associated the increase with the abundant cellular proliferation that resulted. These results have not been repeated and the work discounted because multiple applications of croton oil before the hydrocarbon failed to augment the tumour yield. Recently Tannenbaum, Vesselinovitch and Silverstone (1964) have claimed such an augmenting effect after many trials of this latter character, but it is doubtful for various reasons, amongst them those mentioned by Pound and Bell (1962) and others, whether in this type of experiment the slightly augmented tumour yield is in fact due to the phenomenon dealt with above in this paper. However, it will be shown (Pound, as yet unpublished) that a preliminary application of croton oil or acetic acid does in fact augment the tumour yield after application of carcinogenic hydrocarbons and moreover that augmented tumour yield bears the same relationship or an analogous one to the interval between the preliminary and initiating treatments as is found in the case of urethane.

### SUMMARY

Mice were given a standard tumour producing treatment consisting of an administration of urethane followed by twenty weekly applications of croton oil as a promoting agent.

A preliminary application of croton oil a short interval before administration of the urethane increased the tumour yields in the treated areas. There was little effect at an interval of 9 hours or less. At 12 hours the tumour yield increased abruptly, it reached a maximum at an interval of 15 to 18 hours, and returned to normal at about 5 days. A similar increase in the tumour yield was produced by preliminary application of acetic acid.

The relative increase in the tumour yields was similar if the urethane was administered by injection or by application to the skin, and appeared to be independent of the efficacy of the promoting treatment. It was of similar order if the promoting treatment was delayed for 30 weeks, although the actual tumour yields decreased.

Reasons are put forward to suggest that the augmented tumour yield is the result of an increased number of potential tumour forming foci rather than an intensification of the change in the same number of foci, and for the view that the augmenting effect is associated with production of cellular proliferation in the tissue, in particular with the replication of DNA.

The author wishes to thank Dr. H. Silverstone, Reader in Medical Statistics, University of Queensland, for assistance and advice in the statistical analyses.

### REFERENCES

Berenblum, I. and Haran, N.—(1955) *Br. J. Cancer*, **9**, 453.
Berenblum, I. and Haran-Ghera, N.—(1957) *Br. J. Cancer*, **11**, 77.
Berenblum, I., Haran-Ghera, N., Winnick, R. and Winnick, T.—(1958) *Cancer Res.*, **18**, 181.
Boutwell, R. K., Bosch, D. and Rusch, H. P.—(1957) *Cancer Res.*, **17**, 71.
Boyland, E. and Rhoden, E.—(1949) *Biochem. J.*, **44**, 528.
Bryan, C. E., Skipper, H. E. and White, L.—(1949) *J. biol. Chem.*, **177**, 941.
Bullough, W. S.—(1962) *Biol. Rev.*, **37**, 307.
Bullough, W. S. and Laurence, E. B.—(1964) *Symp. zool. Soc. Lond.*, **12**, 1.
Cornman, I.—(1954) *Int. Rev. Cytol.*, **3**, 113.
Cornman, I., Skipper, H. E. and Mitchell, J. H. Jr.—(1951) *Cancer Res.*, **11**, 195.
Graffi, A., Vlamynch, E., Hoffmann, F., and Schulz, I.—(1953) *Arch. Geschwülstforsch.*, **5**, 110.
Haran, N. and Berenblum, I.—(1956) *Br. J. Cancer*, **10**, 57.
Lindsay, D.—(1956) *Rep. Br. Emp. Cancer Campn*, **34**, 372.
Mitchell, J. H. Jr., Hutchison, O. S., Skipper, H. E. and Bryan, C. E.—(1949) *J. biol. Chem.*, **180**, 675.
Mottram, J. C.—(1944) *J. Path. Bact.*, **56**, 181, 391.—(1945) *J. Path. Bact.*, **57**, 265.
Pound, A. W.—(1962*a*) *Br. J. Cancer*, **16**, 246.—(1962*b*) Reports of Scientific Meetings, College of Pathologists of Australia, No. 2, 18.—(1963) *Aust. J. exp. Biol. med. Sci.*, **41**, 73.
Pound, A. W. and Bell, J. R.—(1962) *Br. J. Cancer*, **16**, 690.
Pound, A. W. and Withers, H. R.—(1963) *Br. J. Cancer*, **17**, 460.
Roe, F. J. C.—(1956) *Br. J. Cancer*, **10**, 72.
Roe, F. J. C. and Salaman, M. H.—(1954) *Br. J. Cancer*, **8**, 666.
Salaman, M. H.—(1958) *Br. med. Bull.*, **14**, 116.
Salaman, M. H. and Roe, F. J. C.—(1953) *Br. J. Cancer*, **7**, 472.
Skipper, H. E., Bennett, L. L. Jr., Bryan, C. E., White, L. Jr., Newton, M. A. and Simpson, L.—(1951) *Cancer Res.*, **11**, 46.
Tannenbaum, A., Vesselinovitch, S. D. and Silverstone, H.—(1964) *Cancer Res.*, **24**, 361.